# A comparison of gas stream cooling and plunge cooling of macromolecular crystals

**DOI:** 10.1107/S1600576719010318

**Published:** 2019-08-23

**Authors:** Kaitlin Harrison, Zhenguo Wu, Douglas H Juers

**Affiliations:** a Department of Physics and Program in Biochemistry, Biophysics and Molecular Biology, Whitman College, 345 Boyer Avenue, Walla Walla, WA 99362, USA

**Keywords:** cryocooling, gas stream cooling, plunge cooling, mosaicity, annealing, cell volume

## Abstract

Cryocooling for macromolecular crystallography is usually performed via plunging the crystal into a liquid cryogen or placing the crystal in a cold gas stream. These two approaches are compared here for the case of nitro­gen cooling. The results show that gas stream cooling, which typically cools the crystal more slowly, yields lower mosaicity and, in some cases, a stronger anomalous signal relative to rapid plunge cooling. During plunging, moving the crystal slowly through the cold gas layer above the liquid surface can produce mosaicity similar to gas stream cooling. Annealing plunge cooled crystals by warming and recooling in the gas stream allows the mosaicity and anomalous signal to recover. For tetragonal thermolysin, the observed effects are less pronounced when the cryosolvent has smaller thermal contraction, under which conditions the protein structures from plunge cooled and gas stream cooled crystals are very similar. Finally, this work also demonstrates that the resolution dependence of the reflecting range is correlated with the cooling method, suggesting it may be a useful tool for discerning whether crystals are cooled too rapidly. The results support previous studies suggesting that slower cooling methods are less deleterious to crystal order, as long as ice formation is prevented and dehydration is limited.

## Abstract 2

Gas stream cooling at 100 K is found to yield lower mosaicity than plunge cooling into liquid nitro­gen at 77 K for several different protein crystals.

## 1. Introduction   

Diffraction from crystals can be used to determine both structural and dynamical information about macromolecules. However, when macromolecular crystals are exposed to X-ray radiation, they accumulate free radicals that cause degradation of the diffraction data quality over time (Garman & Owen, 2006 ▸). In order to counteract radiation damage, macromolecular crystals are often cryogenically cooled before exposure to X-rays, which slows down the radiation damage by limiting free radical diffusion (Garman & Schneider, 1997 ▸).

However, cooling itself can cause damage to macromolecular crystals, increasing mosaicity, reducing diffraction power and limiting the resolution of the resulting electron density maps. Increases in crystal disorder and mosaicity not only reduce the diffraction limit of the crystal, but also make data collection more difficult by requiring larger crystal-to-detector distances and smaller oscillations. This is particularly acute for crystals with large unit cells and for Laue diffraction [the method of choice for neutron crystallography (Langan & Greene, 2004 ▸)], which produce diffraction patterns with closely spaced Bragg diffraction spots.

Most approaches to limiting cooling-induced damage are based on preventing ice formation by cooling fast enough to reach the glass transition temperature before ice crystallization can start. This can be achieved either by increasing the cooling rate (Teng & Moffat, 1998 ▸; Warkentin *et al.*, 2006 ▸) or by reducing the ice nucleation rate with cryoprotective agents (Warkentin *et al.*, 2013 ▸). In addition to ice prevention, the thermal contraction of the cryosolvent is an important parameter involved in cooling-induced damage of some crystals (Juers *et al.*, 2018 ▸).

Several different methods used to cool macromolecular crystals have been described, with different effects on the cooling rate. The two most common approaches are to plunge the crystal into liquid nitro­gen at 77 K or to place the crystal in a cold nitro­gen gas stream at 100 K (Hope, 1988 ▸; Teng, 1990 ▸). However, there has been little systematic work to determine if one of these approaches yields higher-quality diffraction than the other. In some cases, publications do not describe which approach was used, which can make reproducing experiments potentially problematic.

Here we investigate different cooling methods for several different protein crystals. We find that as long as ice formation is prevented and care is taken to limit damaging dehydration effects, gas stream cooling yields lower mosaicity than more rapid cooling methods (plunge cooling and hyperquenching). The anomalous signal can also be affected. In our analysis below, we consider possible physical explanations for the observed differences, and for the similarity between protein structures from plunge cooled and gas stream cooled crystals. We also describe approaches for recognizing, limiting and recovering plunge cooling sourced crystal disorder.

## 2. Methods   

### 2.1. Reagents and crystals   

All reagents were purchased from Sigma–Aldrich (St Louis, Missouri, USA), with protein catalog numbers as follows: thermolysin = P1512, lysozyme = L6876, proteinase K = P6556 and α-lactalbumin = L5385. All crystals were grown using hanging drop vapor diffusion in 24-well VDX plates (Hampton Research, Aliso Viejo, California, USA) at room temperature (RT; 295 K unless noted otherwise). For tetragonal thermolysin, the drop composition was 50%(*v*/*v*) thermolysin from *Bacillus thermophilicus* (150 mg ml^−1^) in 45%(*v*/*v*) dimethyl sulfoxide (DMSO), and 50% DMSO [45%(*v*/*v*)] and 1 *M* ZnCl_2_, and the drop size was 6 µl (Hausrath & Matthews, 2002 ▸). The well solution was 1 ml of 1 *M* AmSO_4_. Hexagonal thermolysin crystals were grown as above but without the ZnCl_2_. The other crystals were grown using drops of 6–8 µl of 50% well solution and 50% protein solution. For α-lactalbumin: protein = 30 mg ml^−1^ in water; well = 18–19.5% PEG 8K, 50 m*M* KH_2_PO_4_ (Mueller-Dieckmann *et al.*, 2007 ▸); for proteinase K: protein = 50 mg ml^−1^ in water, well = 20–30% PEG 8K; for lysozyme: protein = 80 mg ml^−1^ in water, well = 5% NaCl, 20 m*M* NaC_2_H_3_O_2_ pH 4.5 (Farley & Juers, 2014 ▸). Crystal sizes were as follows: tetragonal thermolysin 100–500 µm octahedra; hexagonal thermolysin 50–400 µm rods; α-lactalbumin 100–900 µm parallelpipeds; lysozyme 200–500 µm parallelpipeds; proteinase K 50–400 µm chunks.

### 2.2. Crystal handling and mounting   

Crystals were placed on glass coverslips under a humid flow at ∼95% relative humidity (r.h.) (Farley *et al.*, 2014 ▸) using an in-house made humidity control device similar to the Watershed (MiTeGen, Ithaca, New York, USA). Crystals were removed from their mother liquor and transferred to a 15 µl drop of cryoprotective solution, also under humid flow, where they were soaked for 2–3 min. After the soaking, the crystals were mounted with a nylon loop of a diameter matching the crystal size (Hampton Research) so there was some contact between the crystal and the loop. One of four cooling methods was then performed. (1) Gas stream cooling via the vial mounting method (Farley *et al.*, 2014 ▸). Briefly, after soaking, the crystal was mounted in a cryoloop, which was inserted into a cryovial (Hampton Research) containing 500 µl of the cryosolution and with liquid nitro­gen escape holes blocked with clay or tape. The vial was then transferred by hand to the diffractometer and the crystal was mounted directly onto the goniometer with the cryostream in place. As the crystal was placed on the goniometer, the cryovial was immediately removed, leaving the crystal in place to cool in the cryostream. The incubation times in the vial prior to transfer to the goniometer were about 15 s. A 100 K nitro­gen stream was used with sample/shield flow rates of 6 l min^−1^/4 l min^−1^, respectively (Cryojet, Oxford Instruments, Abingdon, UK). (2) Slow plunge cooling using a dewar of liquid nitro­gen and moving slowly (∼3–15 s) through a 1–3 cm-thick cold gas layer into the liquid nitro­gen below (the speed through the cold gas layer and entering the liquid is ∼0.1–1 cm s^−1^). (3) Normal plunge cooling using a dewar of liquid nitro­gen and moving quickly through a 1–3 cm-thick cold gas layer into the liquid nitro­gen below (the speed through the cold gas layer and entering the liquid is ∼100 cm s^−1^). Throughout, unless otherwise specified, protocol (3) was used for plunging. (4) Hyperquenching using a dewar of liquid nitro­gen and a small fan to blow away the 1–3 cm-thick cold gas layer above the liquid surface (Warkentin *et al.*, 2006 ▸) (in principle, there is no cold gas layer and the speed entering the liquid is ∼100 cm s^−1^). Methods (2)–(4) all employed hand plunging using a CryoWand (Hampton Research) with transfer to the goniometer for diffraction data collection using tongs.

In some cases, a fifth protocol was executed to anneal the crystals as follows: (5) Crystals were unmounted directly from the gas stream into a vial containing 500 µl of the crystal cryobuffer, incubated in the vial for ∼1–3 min at RT, and remounted in the gas stream. Controlling the local environment of the crystal in this way improves the reproducibility of annealing compared with conventional annealing in which the cryostream is blocked for a few seconds with the crystal in place (Farley *et al.*, 2014 ▸).

### 2.3. X-ray data collection and processing   

X-ray data were collected using an Oxford Diffraction Xcalibur X-ray diffractometer with a Nova microfocus Cu *K*α X-ray source and Onyx detector (Rigaku Americas, The Woodlands, Texas, USA) at 50 kV, 0.8 mA. The beam divergence was 5.2 mrad (0.30°). The crystal-to-detector distances were 60–70 mm and the target resolution was set to 2.0 Å. Low-temperature (LT; 77 or 100 K, depending on the context) data were collected at 100 K using a nitro­gen gas stream. RT data were collected without a capillary by mounting a crystal directly from the drop onto a micromesh (MiTeGen) and holding the crystal in humid flow at 98.5% r.h., 7.5 l min^−1^, through a 1.9 cm-diameter nozzle positioned about 1 cm from the crystal. In each case, a pre-experiment was conducted with 2 × 6 (0.5°; 20 s) frames separated by 90°. The pre-experiment outputs estimates of cell parameters and mosaicities. In some cases, additional data were then collected and integrated in *CrysAlisPro*, yielding post-refined crystal parameters (Rigaku Oxford Diffraction, 2019 ▸). See below and figure captions for more details.

In *CrysAlisPro*, the ‘mosaicity’ is given in three components, *e*_1_, *e*_2_ and *e*_3_, which are the mosaicities in three directions defined in a coordinate system local to each reflection. *e*_1_ and *e*_2_ are the mosaicities (*i.e.* the angles subtended by the diffraction spots about the origin of the Ewald sphere) in two orthogonal directions tangent to the Ewald sphere (on the image, *e*_2_ is the mosaicity along the direction radial from the beam center). *e*_3_ is the mosaicity in a direction perpendicular to *e*_1_ and *S* − *S*_0_, which is roughly the mosaicity in the scanning direction, where *S* and *S*_0_ are the scattered and incident X-ray vectors, respectively (Kabsch, 2001 ▸).

The *e*_3_ mosaicity parameter is similar to the Reflecting_Range parameter in *XDS*. For the crystals tested here, the *e*_3_ values are about six times greater than the Reflecting_Range estimated standard deviation (e.s.d.), which is the value reported by *XDS* as the mosaicity (Kabsch, 2010 ▸). The *e*_1_ and *e*_2_ mosaicity parameters are similar to the Beam_Divergence parameter in *XDS*.

For structure determination, data were integrated and scaled in *CrysAlisPro* and merged using *AIMLESS* (Evans, 2006 ▸) and *Ctruncate* (French & Wilson, 1978 ▸; Winn *et al.*, 2011 ▸). Structures of tetragonal thermolysin cryoprotected with 50% xylose were determined using normal plunging and gas stream cooling [*i.e.* protocols (3) and (1) above]. The crystals were similar in size (edges of 0.37–0.45 mm), and the data were collected with identical detector distances (70 mm), oscillation ranges (0.5°), target resolutions (1.4 Å) and exposure times (75 s per frame). The *e*_3_ mosaicities were 0.67 and 0.61° for the plunge and gas stream cooled methods, respectively. The data sets were of similar redundancy and quality (Table S1 of the supporting information). Both structures were determined via single-wavelength anomalous diffraction (SAD) in *PHENIX* (Adams *et al.*, 2010 ▸). Each structure was autobuilt in *PHENIX* and improved separately with multiple rounds of model building in *Coot* (Emsley *et al.*, 2010 ▸) and refinement in *PHENIX*. Multiple side chain conformations were built for each structure separately, and towards the end of building, the structures were compared side by side against their *F*_o_ − *F*_c_ and 2*F*_o_ − *F*_c_ electron density maps calculated with *PHENIX*. *F*_o_ − *F*_o_ electron density maps were also calculated with *PHENIX*, using observed amplitudes from the plunge cooled and gas stream cooled crystals and phases from the gas stream cooled structure. Default parameters were used, in which two sets of observed amplitudes are scaled together, but otherwise the maps were unweighted. *Ringer* was used to analyze subtle differences in side chain torsion angle distributions (Lang *et al.*, 2010 ▸). Coordinates and structure factors have been deposited in the Protein Data Bank (codes 6n4w and 6n4z for gas stream and plunge cooled, respectively).

For SAD efficiency studies, crystals of tetragonal thermolysin were used with one of three cryoprotective agents [50% xylose, 50% MPD (2-methyl-2,4-pentane­diol) or 50% DMF (*N*,*N*-di­methyl­formamide)]. Crystals were plunge cooled [protocol (3)], gas stream cooled [protocol (1)] or annealed in-vial [protocol (5)]. The X-ray data were collected with identical detector distances (70 mm), oscillation ranges (0.5°), target resolutions (2.0 Å) and exposure times (30 s per frame). Data collection strategies were determined with *CrysAlisPro* assuming Friedel pairs were not equivalent with 10-fold (xylose and MPD) or 15-fold (DMF) anomalous redundancy (*i.e.* 20-fold or 30-fold overall redundancy assuming Friedel’s law holds). To assess the impact of redundancy on SAD efficiency, data sets were truncated at lower redundancy levels. Data were integrated and scaled in *CrysAlisPro* and merged using *AIMLESS* (Evans, 2006 ▸) and *Ctruncate* (French & Wilson, 1978 ▸; Winn *et al.*, 2011 ▸), or left unmerged for analysis in *SHELX* (Usón & Sheldrick, 2018 ▸). For analysis in *PHENIX*, the truncated data sets were first analyzed with *Xtriage*. *Autosol* was then executed, which consists of a heavy atom search using HYSS (Grosse-Kunstleve & Adams, 2003 ▸), calculation of phases and refinement of heavy atoms using *Phaser* (McCoy *et al.*, 2007 ▸), solvent flattening using *Resolve* (Terwilliger, 2002 ▸), autobuilding, and initial model refinement. For HYSS, ten sulfur atoms were searched for using the resolution limit of the anomalous signal given by *Xtriage*, or 3.0 Å, whichever was lower resolution. The metrics used to assess the anomalous phasing were CC_1/2-anom_ from *AIMLESS*, the final figure of merit (FOM) from *Phaser* and the Rfree of the autobuilt model. The substructure search was also carried out in *SHELX*, in which case the data were initially analyzed in *SHELXC*. The substructure search for ten sulfur atoms was then carried out in *SHELXD* using the resolution limit of the anomalous signal suggested by *SHELXC* (using a cutoff of CC_1/2-anom_ < 0.25), or 3.0 Å, whichever was lower resolution. The indicators used in this case were the plots of CC_all_ versus CC_weak_, the number of tries to find a solution and the pattern of occupancies in the heavy atom sites found. Executions of *SHELXD* were judged to yield successful solutions if the distributions of CC_all_ and CFOM were multimodal, if the plots of CC_all_ versus CC_weak_ indicated groups of tries with higher values separated from a base level group with lower values (*i.e.* in the upper right corner of the plot), and if the plot of occupancy versus heavy atom site number in decreasing occupancy of the top solution indicated some breaks rather than varying smoothly. In some cases, the top solution from *SHELXD* was used in *SHELXE* for calculation of phases, density modification and autobuilding of an initial polyalanine model. The correlation coefficient between the X-ray data and the autobuild trace (CC_partial) was used to assess the quality of the solution from *SHELXE*.

## 3. Results   

### 3.1. For tetragonal thermolysin, the cooling method can impact crystal order and cell volume   

**Figure 1:**
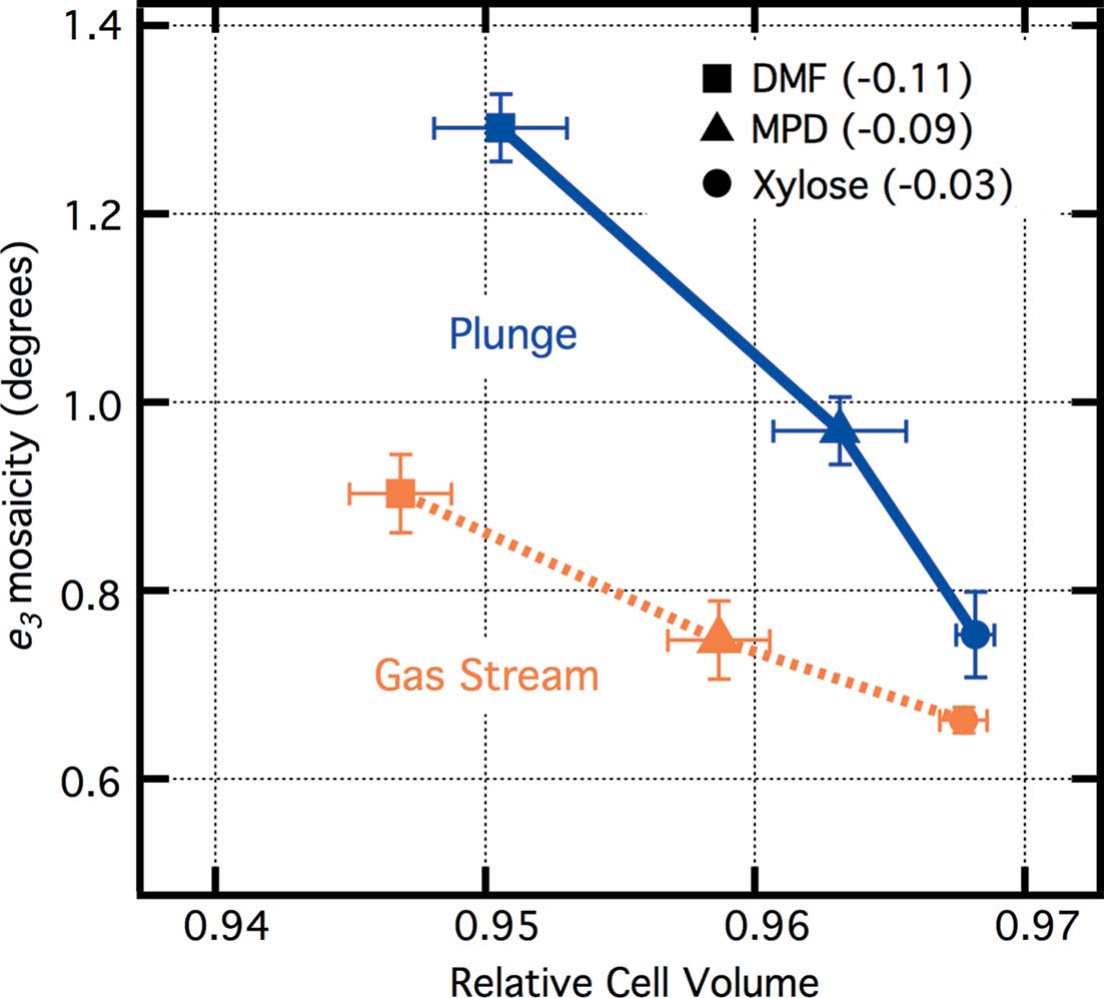
Average *e*_3_ mosacity versus cell volume (relative to RT) of tetragonal thermolysin crystals, comparing plunge cooling into liquid nitro­gen at 77 K with gas stream cooling at 100 K. In each case, cryosolvents of 50%(*w*/*w*) cryoprotective agent with water were used. The fractional change in specific volume of the cryosolvent with cooling between RT and 77 K is shown in parentheses. Greater contracting cryosolvents produce both smaller cell volumes and larger mosaicities (Juers *et al.*, 2018 ▸). Plunge cooling yields more disorder and slightly larger cell volumes than gas stream cooling. In all cases, no ice was observed in the diffraction pattern. For each data point, 3–11 crystals were tested, and the error bars are standard errors. Mosaicities and cell volumes are based on pre-experiments (see *Methods* [Sec sec2]). The plunge cooling procedure used is protocol (3) in Section 2.2: normal plunge cooling using a dewar of liquid nitro­gen and moving quickly through a 1–3 cm-thick cold gas layer into the liquid nitro­gen below.

**Figure 2:**
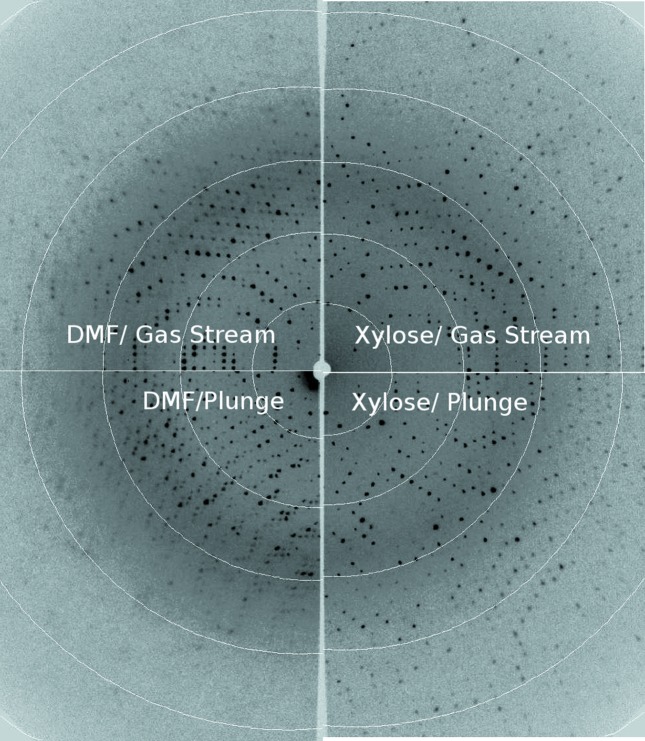
Composite image showing representative diffraction from cryocooled crystals of tetragonal thermolysin for four different conditions. Measured *e*_3_ mosaicities were as follows: xylose and gas stream = 0.6°, xylose and plunge = 0.7°, DMF and gas stream = 0.9°, DMF and plunge = 1.9°.

Fig. 1 ▸ shows that, for tetragonal thermolysin crystals, plunge cooling into liquid nitro­gen at 77 K yielded higher mosaicities than gas stream cooling at 100 K. Additional tests using variations of plunging showed that removal of the cold gas layer above the liquid nitro­gen had little effect on the mosaicity, whereas moving the crystal slowly through the cold gas layer yielded slight mosaicity reductions in comparison with normal plunge cooling (Table 1 ▸). We can therefore summarize the mosacity results approximately as follows:
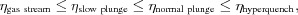
where η is the crystal mosaicity (Bellamy *et al.*, 2000 ▸). The spread between the highest and lowest mosaicities is smaller for less contractile cryosolvents. It is noteworthy that these results would not be expected to hold if cryoprotectant conditions are insufficient to prevent ice formation with all conditions. Cell volumes also depended on the cooling method, with plunging yielding up to 0.5% larger cell volumes (Fig. 1 ▸ and Table S2). Fig. 2 ▸ shows example diffraction patterns for a subset of the conditions from Fig. 1 ▸, illustrating the range of diffraction quality observed.

The above tests were carried out on crystals with linear dimensions of 300–500 µm. Smaller crystals (100–200 µm) were also tested with DMF and MPD as cryoprotectants, showing similar mosaicities but smaller cell volumes compared with the larger crystals (Tables 1 and S1).

### 3.2. The resolution dependence of the reflecting range depends on the cooling method   

**Figure 3:**
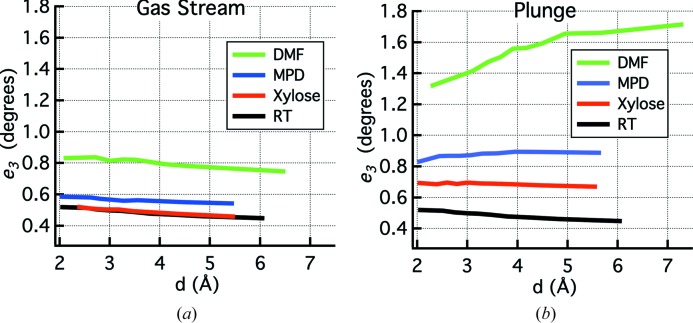
Plots of *e*_3_ mosaicity versus resolution, *d*, for crystals of tetragonal thermolysin gas stream cooled and plunge cooled with different cryoprotective agents. (*a*) Comparison of gas stream cooled crystals [protocol (1), Section 2.2] with an RT crystal. (*b*) Comparison of plunge cooled crystals [protocol (3), Section 2.2] with an RT crystal. The upward shift relative to RT indicates greater strain or angular spread of domains, whereas slopes that are less negative or positive suggest a smaller domain size. The plots indicate that plunge cooling tends to produce a smaller domain size than gas stream cooling, as well as increased variation in unit-cell dimensions and/or domain orientations. Data are based on 2 × 50 0.2° frames roughly separated by 90°, with exposure times of 12–16 s per frame. Each plot is the average of the two 50-frame runs and within the particular resolution bin being plotted. Note that the smaller oscillation width (0.2°) than the other experiments (0.5°) reduces *e*_3_, so the *e*_3_ values here and in Table 2 ▸ are not comparable to *e*_3_ values in other graphs and tables. Additionally, the relatively large beam divergence of our in-house X-ray source will mask small increases in crystal disorder (*i.e.* gas stream cooled xylose soaked crystals are likely to be more disordered than the RT crystal, although they have nearly identical parameters).

To learn more about the nature of the cooling induced damage, we studied the diffraction spot profiles as a function of resolution, which can provide information about the type of disorder present in the crystal using the mosaic block model (Nave, 1998 ▸; Juers *et al.*, 2007 ▸). An example is shown in Fig. 3 ▸ in which the *e*_3_ mosaicity is plotted versus resolution. For RT crystals this plot has a negative slope, while gas stream cooling mostly shifts the plot upwards [Fig. 3 ▸(*a*)]. Normal plunge cooling, on the other hand, shifts the plot upwards and increases the slope [Fig. 3 ▸(*b*)]. We examined these plots for several different crystals, finding gas stream cooling biased the plots towards negative slopes whereas normal plunge cooling biased them towards positive slopes (Table 2 ▸). The *e*_3_ parameter corresponds to the reflecting range, and the slope of *e*_3_ versus *d* is controlled by both the wavelength dispersion of the incident X-ray beam (δλ/λ) and, within the domain model, the domain size (*s*) (Nave, 1998 ▸; Juers *et al.*, 2007 ▸). A negative slope indicates a large domain size, allowing the plot to be dominated by the wavelength dispersion. A positive slope, on the other hand, indicates a smaller domain size sufficient to overcome the effects of the wavelength dispersion. Normal plunge cooling therefore appears to reduce the domain size more than gas stream cooling. Note that, in all cases, the upward shift, which is greater for plunge cooling, is indicative of increased variation of cell parameters (strain) and/or an increased angular spread of domains.

### 3.3. SAD efficiency and gas stream annealing of plunge cooled tetragonal thermolysin crystals   

**Figure 4:**
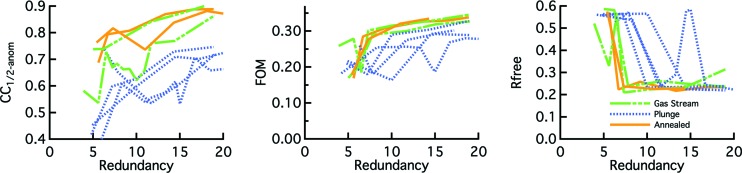
Metrics for SAD structure determination for 50% MPD soaked thermolysin crystals. CC_1/2-anom_ is for the low-resolution bin (∼30–8 Å) from *AIMLESS* (Karplus & Diederichs, 2015 ▸; Evans, 2006 ▸). FOM is the figure of merit from *PHENIX* using *Autosol* (Read & McCoy, 2011 ▸; Adams *et al.*, 2010 ▸). Rfree is for the autobuilt model in *PHENIX* without any user intervention. Metrics are plotted against the overall redundancy of the data set to 2.0 Å, which corresponds to roughly 2× the anomalous redundancy. Blue dots = plunge cooled [protocol (3)]; solid orange = gas stream cooled [protocol (1)]; green dash = plunge cooled followed by rewarming and recooling in the gas stream [protocol (5)].

We also examined the efficiency of SAD structure determination. In this case, the metrics are more dependent on the data collection procedure than for the mosaicity, but some trends are clear. Gas stream cooling yielded a stronger anomalous signal and SAD structures could be determined more efficiently (*i.e.* with lower redundancy data) than with plunge cooling. As with the mosaicity, the effects were more noticeable for greater contracting cryosolvents (Figs. 4 ▸ and S1–S4 of the supporting information).

Recovery of lost order in plunge cooled tetragonal thermolysin crystals was tested by unmounting the crystal into a vial containing the appropriate cryosolution, letting it equilibrate for about 1–3 min at RT and then remounting in the cold gas stream [protocol (5); see *Methods* [Sec sec2]]. This procedure reduced mosaicity and improved the anomalous signal. For DMF soaked crystals, SAD structures could not be determined after plunge cooling [protocol (3)] at 30-fold redundancy for three of the four crystals, but the vial-based annealing procedure yielded successful SAD structures with as little as 10-fold redundancy. All plunge cooled MPD soaked crystals yielded successful SAD structures, but annealing reduced the redundancy required for a solution by a factor of ∼2. See supporting information for more details.

The relationship between the annealing procedure and the domain structure was examined with *e*_3_ versus *d* plots (Fig. S5). In the case of DMF, annealing of plunge cooled crystals allows the domain structure to recover to a more highly ordered state than is achieved by crystals only subjected to gas stream cooling.

### 3.4. Effects on the protein structure   

**Figure 5:**
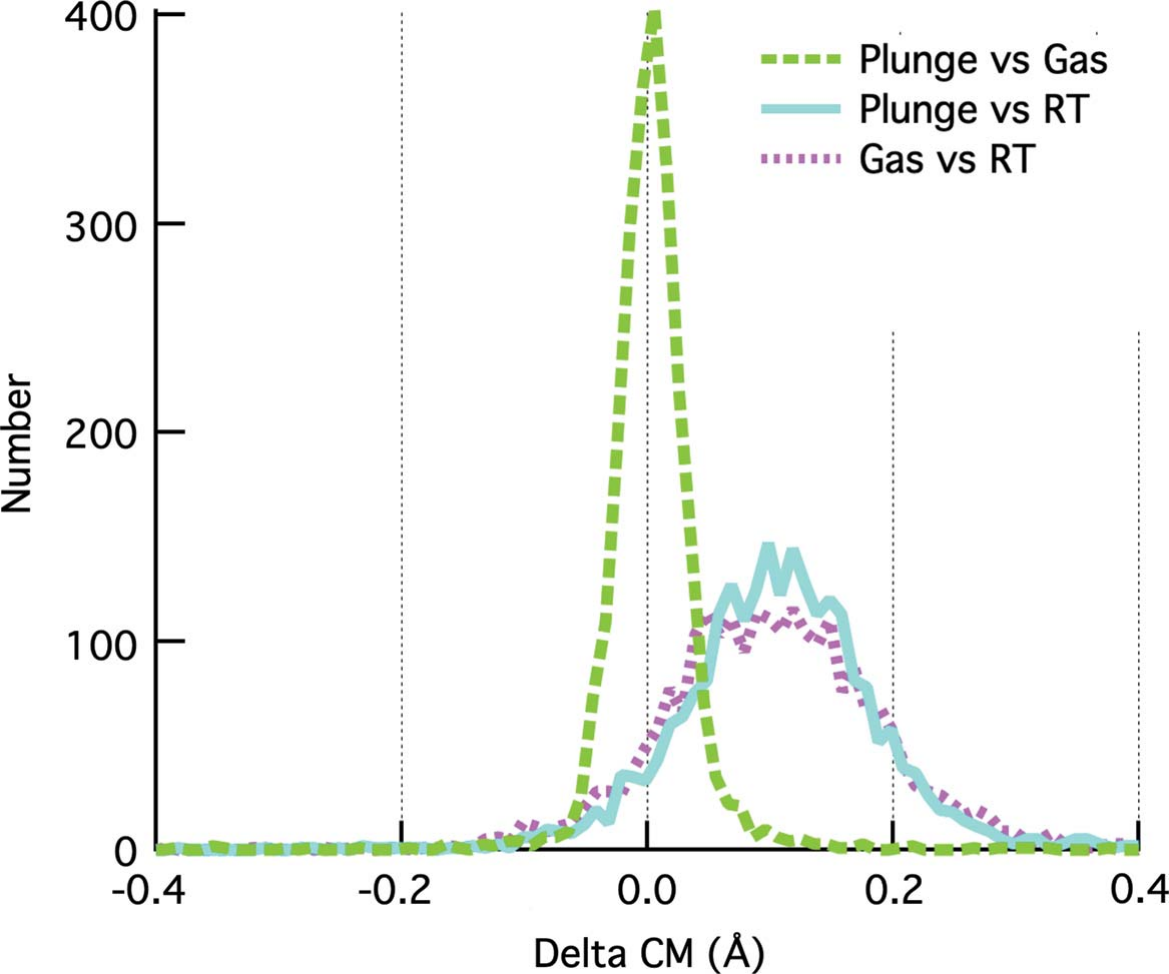
Main chain structural similarity for tetragonal thermolysin cooled with different methods in the presence of 50% xylose. The plots show distributions of the differences (Delta) in distances from the center of mass (CM) for every Cα in the pair of structures (Frauenfelder *et al.*, 1987 ▸). The LT versus RT distributions show an average positive value since the LT structures have smaller distances due to thermal contraction. The distributions versus RT are similar for plunge and gas stream cooling. The distribution for the LT plunge versus LT gas stream conformations is about 3–4× narrower than the LT versus RT distributions. Considered together these results suggest that the two cooling methods have very similar impacts on the main chain conformation of the protein. The RT structure was PDB entry 5un3 (Juers *et al.*, 2018 ▸), for which X-ray data were collected on a crystal soaked in 50% xylose. The LT structures used in the plots are reported here and differ in cell volume by 0.1%. Using the previously reported gas stream cooled structure for 50% xylose (PDB entry 5uua; Juers *et al.*, 2018 ▸), which differs in cell volume from the two LT structures reported here by 0.1–0.2%, gives similar results.

To assess the impact of the cooling method at the individual protein level, structures were determined of normal plunge cooled and gas stream cooled tetragonal thermolysin. The cryoprotectant used was 50% xylose, which produces the smallest cell volume differences between plunge and gas stream cooling (here a 0.07% volume difference, Table S2). Keeping the unit cells similar should reduce the effects of differential crystal packing interactions. The differences between the refined protein structures are subtle. The Cα root-mean-square deviation (RMSD) is 0.05 Å, and the hinge bending angle between the two structures is 0.2°. For reference, the Cα RMSD between two different RT structures soaked in different solutions (50% MPD versus 50% xylose) was 0.09 Å, and hinge bending angles between different thermolysin structures have been observed up to 5° (Hausrath & Matthews, 2002 ▸; Juers *et al.*, 2018 ▸). Main chain configurations were also probed using delta center-of-mass plots (Frauenfelder *et al.*, 1987 ▸), which suggest the two cooling methods have very similar effects on the overall protein configuration (Fig. 5 ▸). The *F*_o_ − *F*_c_ electron density maps are very similar, with many blobs appearing nearly identical for the two structures (not shown). The highest peaks in the *F*_o_ − *F*_o_ electron density maps are for small shifts of bound metal ions probably associated with the slight non-isomorphism between the two crystals. Both structures have the same 33 residues built with alternative side chain conformations, whose occupancies are highly correlated, with the largest occupancy difference being 0.1 (Fig. S6). This can be compared with occupancy shifts between RT and cryocooled structures, in which about 1/3 of alternative side chain conformations cause occupancy shifts greater than 0.2 (Fraser *et al.*, 2011 ▸). More subtle differences in side chain conformations were probed by comparing *Ringer* plots of electron density variation about Chi-1. The correlation between these plots is very high, with more than 93% of the residues showing a Pearson correlation of their *Ringer* plots greater than 0.9 (Fig. S7). Cooling shifts the distribution, consistent with previously described modulation of side chain conformational distributions from cooling (Fraser *et al.*, 2011 ▸). However, the shifts in the distribution are about the same for plunge and gas stream cooling, and the resulting two cryocooled structures have a high degree of similarity.

### 3.5. Tests on other crystals   

Plunge cooling yielded higher mosaicities than gas stream cooling for other crystal systems (Table 3 ▸). Including tetragonal thermolysin, there were nine crystal/cryoprotectant combinations tested (Tables 2 and 3). In each case the average plunge cooled mosaicity was higher than the average gas stream cooled mosaicity. In six of the nine cases, the mosaicity of every plunge cooled crystal was higher than the mosaicity of every gas stream cooled crystal (the exceptions being tetragonal thermolysin with 50% xylose or 30% MPD, and tetragonal lysozyme).

## 4. Discussion   

### 4.1. Cooling method effects on crystal order and cell volume   

Cryogenic cooling of macromolecular crystals generally increases disorder severalfold over the RT state (Bellamy *et al.*, 2000 ▸). For the crystals investigated here, normal plunge cooling produces greater crystal disorder (as assessed from the mosaicity) than gas stream cooling. There are multiple ways in which these two approaches may affect cryocooling differently, including the cooling rate and susceptibility to dehydration.

The average cooling rate between RT and LT is higher for ‘normal’ plunge cooling (*i.e.* plunging with speeds of 50–100 cm s^−1^), although gas stream cooling may be faster down to ∼250 K (Teng & Moffat, 1998 ▸). The higher cooling rate is due to both a larger heat transfer coefficient (∼10× greater for liquid N_2_ versus gaseous N_2_) and a larger temperature difference (∼1.1× greater for liquid N_2_ at 77 K versus gaseous N_2_ at 100 K) (Kriminski *et al.*, 2003 ▸). There have been multiple studies suggesting that the cooling rate may impact diffraction quality for particular samples. Very slow cooling (0.1 K s^−1^) was observed to reduce the mosaicity of thaumatin crystals by a factor of 2 over plunge cooling (Warkentin & Thorne, 2009 ▸). Other strategies for slow cooling have included plunge cooling to 150 K (Luger *et al.*, 1997 ▸; Sargent & Richmond, 2004 ▸; Edayathumangalam & Luger, 2005 ▸) or cooling in capillaries to 100 K (Yao *et al.*, 2004 ▸; Fujimoto *et al.*, 2009 ▸), all of which improved diffraction over normal plunge cooling into liquid nitro­gen. On the other hand, plunge cooling into liquid propane was found to yield higher quality diffraction than gas stream cooling for T7 DNA crystals (Garman & Doublié, 2003 ▸). Undoubtedly there are many unreported examples of one approach being favored over another.

Dehydration is known to impact crystal order. Many studies have shown there can be both improvement and reduction of order from both controlled and unintentional dehydration during crystal mounting (Kiefersauer *et al.*, 2000 ▸; Russi *et al.*, 2011 ▸). Here we have used procedures designed to limit dehydration during crystal mounting. First, crystals were manipulated under humid flow (r.h. ≃ 95%). In comparison with the ambient r.h. in our laboratory (10–50%), this reduces the rate of dehydration from drops and crystals by ≳10×. For gas stream cooling, we employed a vial mounting procedure to transfer the crystal directly to the cold gas stream rather than moving through the low-humidity ambient air (Farley *et al.*, 2014 ▸). For plunge cooling, dehydration during transfer was limited by moving the crystal as quickly as possible through a very short distance to the dewar of liquid nitro­gen. We are therefore relatively certain that the observed differences between gas stream cooling and plunge cooling are due to events occurring after the temperature change of the crystal has started.

Cooling-induced damage can in principle include both non-homogeneous and homogeneous processes (Kriminski *et al.*, 2003 ▸). The former involve the creation of temperature gradients during rapid cooling, so faster cooling during plunging should increase non-homogeneous damage, and this should be greater for larger cell contractions, as we observe. Non-homogeneous damage may involve stress created from mismatched thermal contractions of cryosolutions and crystal pores and may occur even with very slow cooling (Juers & Matthews, 2001 ▸; Kriminski *et al.*, 2002 ▸; Warkentin & Thorne, 2009 ▸; Juers *et al.*, 2018 ▸). This stress may create flow of solvent within solvent channels that accumulates in defect regions, breaking the crystal into smaller domains during cooling. The distance traveled by the solvent within the channels can be characterized by a length scale *L*_crit_ = *R*[Δ*t*/(4κη)]^1/2^, where *R* is the channel radius, 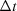 is the time of solvent flow, and κ and η are the compressibility and viscosity of the solvent within the channels, respectively (Juers *et al.*, 2018 ▸). Solvent transport during cooling due to pore pressurization should be roughly less than this length, because longer distances would require so much pressure that the solvent will compress instead. *L*_crit_ may therefore be related to the domain size after cooling. Faster cooling would reduce Δ*t*, causing *L*_crit_ and thus the domain size to be smaller, increasing mosaicity.

Removal of the cold gas layer above the liquid surface [protocol (4)] can increase the cooling rate during plunging by limiting cooling in the gas phase above the liquid (Warkentin *et al.*, 2006 ▸; Berejnov *et al.*, 2006 ▸). Here cold gas layer removal had little effect on the mosaicities of xylose and DMF soaked tetragonal thermolysin crystals. It is likely that our combination of plunge speeds (50–100 cm s^−1^), sample sizes (300–500 µm edges) and cold gas layer thicknesses (1–3 cm) caused most of the cooling during normal plunging to occur in the liquid rather than in the cold gas layer, in which case cold gas layer removal would have little effect on the cooling rate.

On the other hand, reducing the plunge speed to <1 cm s^−1^ [protocol (2)] reduced the mosaicities relative to normal plunging. At this speed, even for our relatively large samples, most of the cooling probably occurs in the cold gas layer (Warkentin *et al.*, 2006 ▸; Berejnov *et al.*, 2006 ▸), reducing the cooling rate. The cryoprotectant concentration is high enough to prevent ice formation, and temperature-induced relaxations have a chance to occur more evenly throughout the crystal.

For smaller crystals, temperature gradients in the crystal during plunging are smaller, which should reduce non-homogeneous damage (Kriminski *et al.*, 2003 ▸). However, because cooling times are shorter (*i.e.* smaller 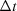), homogeneous damage as described above should be amplified. Here reducing the crystal linear dimensions by a factor of 2–3 from 300–500 µm to 100–200 µm yielded essentially no change in the mosaicities of plunge cooled MPD and DMF soaked tetragonal thermolysin crystals, suggesting that homogeneous processes are involved in the plunge-cooling-induced damage to these crystals.

Plunge cooling involves transfer to 77 K versus the typical 100 K used for gas stream cooling. The extra 23 K should increase the cooling rate by only about 10% (Kriminski *et al.*, 2003 ▸), but the lower temperature could conceivably access an order–disorder transition that explains the larger mosaicity. The fact that slow plunging through the cold gas layer into liquid nitro­gen can yield similar mosaicities to gas stream cooling suggests that a rate effect is involved, not simply going to the lower temperature. Further experiments with more precise control over the cooling rate and target temperature may be helpful.

One possibility that cannot be discarded is that there may be a directionality to the cooling involved. Infrared images show that gas stream cooling proceeds in a pulse along the direction of the cold flow (Snell *et al.*, 2002 ▸). Cooling during plunging may be more omnidirectional, triggering greater strain.

Plunging produced smaller reductions in cell volume for tetragonal thermolysin than gas stream cooling. A cell volume reduction could be caused by shrinkage of the material present in the cell (due to purely thermal effects or pore pressurization during cooling), or transport of material out of the cell (Juers *et al.*, 2018 ▸). Since the glass transition is a kinetic phenomenon, its properties are governed by the cooling rate (Jones, 2002 ▸). With most liquids, faster cooling yields a higher glass transition temperature and smaller density increases. Here this would lead to larger volumes with plunge cooling. Confirming whether a factor of ten in the cooling rate could lead to a ∼1% change in cell volume awaits further experiments, for example by varying the cooling rate in vitrified density measurements of cryosolvents (Alcorn & Juers, 2010 ▸; Shen *et al.*, 2017 ▸). Solvent transport may also explain the cell volume difference, because greater resistance to solvent flow due to faster cooling may limit the total volume of solvent flowing out of any particular unit cell, resulting in smaller unit-cell reductions for plunging.

In summary, the fact that faster cooling yields higher mosaicity is not unexpected as there is ample precedent for this observation as discussed above. However, it is somewhat surprising that just the difference in rate between gas stream and plunge cooling is enough to affect crystal order. Both approaches are executed relatively easily, allowing for simple checking for differences in diffraction quality. Automated crystal harvesting approaches based on gas stream cooling seem well founded (Zander *et al.*, 2016 ▸) as long as adequate steps are taken to limit dehydration (Farley *et al.*, 2014 ▸). Using plunge cooling may offer the additional flexibility of controlling the cooling rate (Warkentin *et al.*, 2006 ▸; Viola *et al.*, 2011 ▸; Deller & Rupp, 2014 ▸) using, for example, the MiTeGen NANUQ, again, as long as dehydration effects are limited.

### 4.2. SAD efficiency and annealing   

SAD efficiency studies were performed with the tetragonal form of thermolysin, a 316 residue protein with a predicted Bijvoet ratio of ∼1% for an X-ray wavelength of 1.54 Å (see supporting information). A rough estimate of the required signal-to-noise ratio for successful structure determination via SAD with this relatively low Bijvoet ratio is ∼70 (Olczak & Cianci, 2018 ▸). In practice, overall 〈*I*/σ〉 levels of 20–30 were required for solutions for most crystals, but this took different levels of redundancy for different cryoprotectants and protocols. The three DMF soaked plunge cooled crystals that did not yield solutions only achieved 〈*I*/σ〉 levels of 8–13 at 30-fold redundancy. Because crystal decay from radiation damage limits the benefit of collecting higher-redundancy data, even at lower intensity in-house X-ray sources (Sarma & Karplus, 2006 ▸), gas stream cooling or plunge cooling accompanied by moving slowly through the cold gas layer may require fewer crystals to achieve a structure solution than normal plunge cooling, especially with certain cryoprotective agents.

Diffraction spot broadening from increased crystal disorder with cooling will reduce the accuracy of the intensity measurement, requiring higher redundancy to achieve the same accuracy in comparison to a more well ordered crystal. In addition, internal non-isomorphism from unit-cell variation may reduce the anomalous signal. Comparing a DMF plunge cooled crystal with an RT crystal, we see a ∼0.7° (0.012 rad) increase in the intercept of *e*_3_ versus *d* (Table 2 ▸), suggesting an upper limit for cell edge variation of 1.2% (or ∼1 Å for tetragonal thermolysin) within the plunge cooled crystal (Juers *et al.*, 2007 ▸). This internal non-isomorphism appears to exceed the less than 1 Å cell parameter variation observed within data set clusters used for successful multicrystal sulfur SAD structure determination of four different proteins (Giordano *et al.*, 2012 ▸).

Annealing of plunge cooled crystals reduced the redundancy required for structure determination. This was particularly effective for DMF, in which case the plunged-then-annealed crystals had lower mosaicities than crystals just cooled in the gas stream (Table S4). All three plunged DMF soaked crystals that did not yield solutions produced solutions after annealing with redundancy levels of 10–15.

Plots of *e*_3_ versus *d* (Fig. S5) suggest that annealing increases the domain size and reduces unit-cell variation and/or domain orientational variation compared with the originally plunge cooled crystal. Interestingly, the one plunge cooled DMF crystal that yielded a structure solution had relatively high *e*_3_ values, but the lowest slope of *e*_3_ versus *d*, implying a larger domain size and pointing to damage processes involving reduced domain size as problematic for SAD structure determination.

### 4.3. Effects on protein conformation   

The predicted cooling time for plunge cooling into liquid N_2_ is about 10× smaller than for gas stream cooling (Kriminski *et al.*, 2003 ▸), which could impact quenching of protein conformations differently (Halle, 2004 ▸) and the resulting side chain and main chain conformational distributions present at LT (Juers & Matthews, 2004*b* ▸; Fraser *et al.*, 2011 ▸). To investigate this possibility, we compared plunge cooled and gas stream cooled structures of tetragonal thermolysin with 50% xylose as cryoprotectant. Although, as has been studied by many authors (*e.g.* Hartmann *et al.*, 1982 ▸; Frauenfelder *et al.*, 1987 ▸; Earnest *et al.*, 1991 ▸; Rasmussen *et al.*, 1992 ▸; Tilton *et al.*, 1992 ▸; Kurinov & Harrison, 1995 ▸; Juers & Matthews, 2001 ▸; Fraser *et al.*, 2011 ▸; Atakisi *et al.*, 2018 ▸), cryocooling had measurable effects on the protein structure, the resulting plunge cooling and gas stream cooled structures are very similar to each other at both the main chain and side chain levels (see Section 3.4, Figs. 5 ▸ and S6–S7). This contrasts with a recent report suggesting that cryocooling may induce conformational heterogeneity such that two LT structures are as different from each other as they are from the starting RT structure (Fischer *et al.*, 2015 ▸). However, in that case the two LT crystals were more non-isomorphous (*i.e.* 7% difference in unit-cell volume), perhaps complicating the comparison.

The most dissimilar side chain electron densities (judging from *Ringer* Chi-1 plot correlations – see supporting information) were for surface exposed side chains with multiple conformations, including two clusters of residues. The first cluster (166, 157, 158 and 159) includes residues that interact with the substrate and the active site zinc. Three of these residues are modeled with two conformations, but between the plunge cooled and gas stream cooled structures the occupancies differ by less than 0.1. The second cluster (181, 182 and 183) forms a part of a turn at the end of a helix and includes an interaction with a bound cation. These three residues are modeled with multiple conformations, including a peptide flip of Lys182 away from the RT configuration, which is modeled with occupancies of 0.33 and 0.26 in the plunge cooled and gas stream cooled cases, respectively.

There is thus very little evidence in this particular case that faster cooling via plunging into liquid nitro­gen changes conformational ensembles present at LT compared with gas stream cooling. Whether larger differences in cooling rate can appreciably modulate main chain and/or side chain conformations (*e.g.* via hyperquenching, liquid propane or using a wide range of crystal sizes) remains an open question.

It has been previously shown that structures determined at LT can be as similar to each other as structures determined at RT (Juers *et al.*, 2018 ▸). However, crystal mounting for LT data collection is subject to variation from dehydration and to variation in contraction if cryosolvents differ (Juers *et al.*, 2018 ▸; Farley & Juers, 2014 ▸), effects which can impact volumes and conformations of both the unit cell and protein (Atakisi *et al.*, 2018 ▸). Dehydration control, which is built into RT mounting methods, is thus equally important for cryocrystallography to ensure that observed structural differences are not caused by differential dehydration during crystal manipulation and mounting.

### 4.4. Choice of type and concentration of cryoprotective agent   

For some crystals the thermal contraction of the cryosolvent is an important parameter that can be adjusted to reduce cooling induced disorder (Juers *et al.*, 2018 ▸). Here this was more important for plunge cooled crystals than gas stream cooled crystals. For tetragonal thermolysin, choosing the right cryoprotective agent limits the advantage of gas stream cooling (Table 1 ▸).

Thermal contraction is also impacted by cryoprotectant concentration. For thermolysin, 30% MPD still yields ice-less diffraction but with about 4–5% solvent contraction in comparison to 8–9% contraction at 50% (Juers & Matthews, 2004*a* ▸; Tyree *et al.*, 2018 ▸). For 30% MPD, the difference in mosaicity between the gas stream cooled and plunge cases is smaller than that at 50% MPD and the cell volumes are more similar (Tables 1 and S1). Limiting the difference in damage between plunge and gas stream cooling can therefore be approached with both cryoprotectant type and concentration.

For many crystals there will be a practical limit to slow cooling; taking ∼30 s to move DMF soaked crystals through a 3 cm-thick gas layer usually resulted in ice formation. There is thus a tension in which faster cooling is needed to limit ice formation, whereas slower cooling gives time for temperature-induced relaxations in crystal packing and solvent distribution to occur more evenly throughout the crystal, improving the quality of diffraction data. The lowest mosaicity will therefore probably be achieved using the slowest cooling rate that prevents the formation of ice. However, precise control of the cooling rate is challenging. Some options include adjusting the flow rate of the cryostream and controlling the speed with which the crystal moves through the cold gas layer above the liquid nitro­gen (Warkentin *et al.*, 2006 ▸).

In summary, the response of the protein and the crystal to cryocooling procedures inevitably involves more than a simple temperature change. Contraction, solvent flow and dehydration are also in play and can cause not only crystal damage but also non-isomorphism, making comparisons of structures to understand the effects of ligand binding or mutations more difficult. Attention to the composition of the cryosolution as well as possible dehydration effects occurring during mounting procedures will therefore improve both the reproducibility and quality of LT crystal structures.

### 4.5. Strategies for cryo-optimization – feedback from the diffraction pattern   

Plotting mosaicity versus resolution can provide information about the crystal order within the domain model. Smaller domains may indicate the crystal was cooled too quickly and could benefit from gas stream cooling, moving more slowly through the gas layer or annealing in the gas stream. If the beam divergence and wavelength dispersion are known, the domain size can be quantified (Juers *et al.*, 2007 ▸; Bellamy *et al.*, 2000 ▸). Incorporation of such calculations into diffraction data integration software would improve general knowledge about changes in crystal order caused by post growth treatments, including cryocooling. Additional information to guide cooling procedures may be available by considering the resolution dependence of the diffracted beam divergence (Nave, 1998 ▸) in addition to the reflecting range.

## 5. Conclusions   

For the nine crystal/cryoprotectant combinations tested here, gas stream cooling at 100 K using the vial mounting approach yielded lower mosaicity than normal plunge cooling into liquid nitro­gen at 77 K. For the subset of crystals tested, the anomalous signal from gas stream cooling was comparable to or stronger than that from plunge cooling. For plunge cooling, in some cases moving the crystal slowly through the cold gas layer above the liquid surface yielded lower mosaicity. Therefore, as long as ice formation is prevented and dehydration is limited, disorder induced from cooling appears to be minimized by cooling more slowly. Finally, considering the resolution dependence of the reflecting range should provide some guidance towards the optimal cooling rate.

## Supplementary Material

Supporting information file. DOI: 10.1107/S1600576719010318/ei5040sup1.pdf

PDB reference: 6n4w

PDB reference: 6n4z

## Figures and Tables

**Table 1:** Mosaicities (°) of cryocooled tetragonal thermolysin crystals For each condition, 3–11 crystals were tested using the pre-experiment protocol with 0.5° oscillations (see *Methods* [Sec sec2]). Uncertainties are the standard errors of the mean. Where two numbers are quoted, the left is for crystals with 300–500 µm edges (as for the other conditions) and the right for crystals with 100–200 µm edges.

Cryoprotective agent	Vial mount (gas stream)	Slow plunge	Normal plunge	Cold gas layer removal
50% xylose	0.66 (1)	0.67 (0)	0.75 (2)	0.72 (1)
50% MPD	0.75 (1)/0.74 (3)	–	0.97 (5)/0.95 (8)	–
50% DMF	0.90 (4)/1.03 (2)	0.95 (2)	1.29 (4)/1.25 (5)	1.16 (9)
30% MPD	0.71 (2)	–	0.74 (1)	–

**Table 2:** Parameters for linear fits of *e*_3_ versus *d* for various cooling schemes for tetragonal thermolysin (tet TLN) crystals In the domain model, the slope is related to the domain size (a less negative or a more positive slope corresponds to a smaller domain size), while a larger intercept indicates greater variation of unit-cell dimensions and/or domain orientations. Oscillation ranges of 0.2° were used; see Fig. 3 ▸ caption.

Crystal/cryoprotectant	Cooling method	*e*_3_ ( 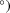	Slope (° Å^−1^)	Intercept ( 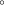 )
Tet TLN	RT	0.49	−0.019	0.56
Tet TLN/xylose	Gas stream	0.50	−0.019	0.56
Tet TLN/xylose	Plunge	0.69	−0.007	0.71
Tet TLN/MPD	Gas stream	0.57	−0.013	0.61
Tet TLN/MPD	Plunge	0.87	0.008	0.85
Tet TLN/DMF	Gas stream	0.80	−0.021	0.89
Tet TLN/DMF	Plunge	1.54	0.068	1.26

**Table 3:** Mosaicities (°) for other cryocooled crystals For each of the first four crystals, 3–6 crystals were tested using the pre-experiment protocol with 0.5° oscillations (see *Methods* [Sec sec2]). Uncertainties are the standard errors of the mean. In each case, the cryoprotectant supplemented the crystallization solution.

Crystal	Cryoprotectant	Gas stream	Plunge cool
Hexagonal thermolysin	50% xylose	0.71 (3)	0.89 (2)
α-Lactalbumin	25% xylose	0.69 (1)	0.87 (2)
Tetragonal lysozyme	40% glycerol	0.85 (4)	0.97 (4)
Proteinase K	4 *M* TMAO†	0.70 (2)	0.79 (2)
Tetragonal thaumatin‡	35% ethyl­ene glycol	0.46 (3)	0.54 (2)

† TMAO is trimethylamine oxide.

‡ Calculated from Table S1 of Farley *et al.* (2014 ▸); *n* = 13 and uncertainties are standard deviations.
