# Supplementary material for: A comparison of gas stream cooling and plunge cooling of macromolecular crystals
Source: J Appl Crystallogr. 2019 Aug 23;52(Pt 5):1222–32. doi: 10.1107/S1600576719010318 (PMC6782077; doi:10.1107/S1600576719010318)

JOURNAL OF
APPLIED
CRYSTALLOGRAPHY

Volume 52 (2019)

Supporting information for article:

A comparison of gas stream cooling and plunge cooling of macromolecular crystals

Kaitlin Harrison, Zhenguo Wu and Douglas H Juers

Assessment of SAD Phasing Efficiency. Tetragonal thermolysin crystals cryocooled with different protocols and cryoprotectants were tested for their ability to yield structures via SAD. Thermolysin is a 316 residue protein that contains two methionine residues (and no cysteines), four calcium binding sites and a catalytic zinc ion. The crystallization buffer included ~ 1 M $ZnCl_2$. Table S3 shows anomalous scattering parameters at 1.54 Å for the likely substructure atoms. The refined structures suggest there are eight primary fully occupied sites – the four calcium sites (three calciums and one zinc, which appears to have displaced one of the calciums based on refined B-factors and occupancies), the catalytic zinc ion, the two methionine sulfur atoms and another zinc ligated to an active site residue, His 231. Additionally, there are several partially occupied sites, including up to three additional sites in the active site, and an apparent zinc-chloride complex spanning His-250 and Lys-238, each residue ligating a tetracoordinate zinc ion. The two zinc ions are bridged by a water molecule and each ligates two chloride ions. The estimated Bijvoet ratio based on the 8 fully occupied sites (3 Ca, 3 Zn, 2 S) is 0.9 % and on 16 fully occupied sites (3 Ca, 7 Zn, 2 S, 5 Cl) is 1.2 % (http://assc.p.lodz.pl/index3.php) (Olczak & Cianci, 2018).

Initially, substructure determination, phasing and automated model building were carried out using the Phenix package (Adams *et al.*, 2010). At the highest levels of data redundancy most of the crystals yielded structures and HYSS/Phaser found 13-19 sites. In the three cases that structures weren't produced, 5-6 sites were found at 30-fold redundancy. As can be seen in Fig S1, in most cases increasing the redundancy improved the anomalous signal (as assessed from $CC_{1/2\text{-anom}}$) and the phases (as assessed from the FOM). On average, these metrics were higher for gas stream cooled crystals than for plunge cooled crystals. This is particularly true for DMF, in which three of the four plunge cooled crystals did not yield structures. However, annealing could restore the diffraction quality to as good or, in the case of DMF, better than gas-stream cooled levels. (Green dash-dot-dot lines in Figs S1, S2 & S5).

The substructure search was also carried out in ShelxD. At the higher levels of redundancy, ShelxD found 8-11 sites. Substructure solutions were found with fewer tries for data sets from gas stream cooled or annealed crystals in comparison with data sets from plunge cooled crystals (Fig. S2). Fig S3 shows examples of output CCall vs CCweak plots from ShelxD, while Fig S4 shows the site occupancies from ShelxD. The three plunge cooled DMF soaked crystals that didn't yield a structure solution in Phenix also didn't yield a solution ShelxD. For these crystals, up to 10,000 tries were executed in ShelxD using the highest redundancy data set, and the top solution was used as input for phasing, density modification and chain tracing in ShelxE. However, the correlation coefficient between the X-ray data and the autobuilt model resulting from ShelxE (CC_partial) never exceeded 12%, whereas for the plunge cooled DMF soaked crystal that did yield a solution, CC_partial was 27% (a correlation of 25% is considered to indicate a successful solution (Uson & Sheldrick, 2018)).

Table S1 X-ray data collection, processing and refinement statistics for tetragonal thermolysin cryoprotected with 50% xylose. Crystal-detector distance = 70 mm, rotation range per image = 0.5°, exposure time = 75 seconds. Space group $P4_12_12$. In both cases X-ray diffraction data were collected at 100 K. CC(work) and CC(free) are correlation coefficients between the observed intensities and intensities calculated from the refined model for the working and test sets respectively.

	Gas Stream Cooled	Plunge Cooled
Resolution range (Å)	30.59 - 1.4 (1.45 - 1.4)	29.4 - 1.4 (1.45 - 1.4)
Unit cell (a, c in Å; volume in Å^3)	96.739, 106.425; 995,973	96.725, 106.535; 996,704
Total reflections	1,167,036 (79,540)	1,145,058 (77,476)
Unique reflections	99,213 (9,652)	98,771 (9,449)
Multiplicity	11.8 (8.2)	11.6 (8.2)
Completeness (%)	99.7 (98.1)	99.2 (96.1)
Mean I/sigma(I)	22.85 (0.79)	20.28 (0.72)
Wilson B-factor (Å^2)	15.6	16.2
R-merge	0.079 (2.62)	0.081 (2.82)
e_3 mosaicity (degrees)	0.61	0.67
R-meas	0.083 (2.79)	0.084 (3.00)
R-pim	0.023 (0.95)	0.024 (1.03)
$CC_{1/2}$	0.999 (0.298)	0.999 (0.277)
CC*	1 (0.678)	1 (0.659)
Mosaicity (degrees)	0.61	0.67
Reflections used in refinement	99,213 (9,598)	98,771 (9,390)
Reflections used for R-free	4893 (457)	4861 (445)
R-work	0.1376 (0.3321)	0.1389 (0.3357)
R-free	0.1569 (0.3702)	0.1612 (0.3636)
CC(work)	0.974 (0.653)	0.974 (0.662)
CC(free)	0.968 (0.613)	0.961 (0.613)
Number of non-hydrogen atoms	3217	3183
macromolecules	2666	2660
ligands	120	100
solvent	431	423
Protein residues	316	316
RMS(bonds - Å)	0.006	0.007
RMS(angles - degrees)	0.86	0.87
Ramachandran favored (%)	96.50	96.18
Ramachandran allowed (%)	3.50	3.82
Ramachandran outliers (%)	0.00	0.00
Rotamer outliers (%)	0.35	0.35
Average B-factor (Å^2)	21.10	21.56
macromolecules	17.80	18.49
ligands	43.63	41.28
solvent	35.21	36.20
PDB Code	6N4W	6N4Z

Table S2 Cell volumes, relative to RT, of cryocooled tetragonal thermolysin crystals. For each condition, 3-11 crystals were tested using the pre-experiment protocol with 0.5-degree oscillations (see Methods). Uncertainties are standard error of the mean. The reference RT volume is taken as 1,033,000 Å^3 (Juers *et al.*, 2018). Where two numbers are quoted, the left is for crystals with 300-500 µm edges (as for the other conditions) and the right for crystals with 100-200 µm edges.

Cryoprotective Agent	Vial Mount (Gas Stream)	Slow Plunge	Normal Plunge	Hyperquench
**50% Xylose**	0.9677 (4)	0.9710 (5)	0.9681 (7)	0.9696 (7)
**50% MPD**	0.9587 (9) / 0.9492 (5)	-	0.9631 (7) / 0.9521 (6)	-
**50% DMF**	0.9468 (19) / 0.9468 (4)	0.9481 (8)	0.9505 (25) / 0.9474 (7)	0.9562 (35)
**30% MPD**	0.9703 (24)	-	0.9698 (10)	-

Table S3 Anomalous scattering parameters for possible substructure atoms (Merrit, 2011). Values are given for f" at λ=1.54 Å.

Element (Z)	f"	K_{edge} (Å)
S (16)	0.56	5.0
Cl (17)	0.71	4.4
Ca (20)	1.30	3.1
Zn (30)	0.69	1.3

Table S4 Cell parameters, mosaicities and e_3 vs d parameters for annealing experiments with tetragonal thermolysin. All values are for crystals at LT (100K). Gas = cooled in the gas stream (protocol 1). Plunge = normal plunge cooling (protocol 3). Anneal = normal plunge then anneal (protocol 5). In the case of xylose, annealing experiments were not performed, but crystal parameters for protocols 1 & 3 are given for reference. For MPD the first two plunge cooled crystals were then annealed, as listed. For DMF, all four plunge cooled crystals were then annealed, as listed. See also Fig. S5. The data in Table S4 are not included in Table 1, although the general trends are similar (i.e. higher mosaicity for plunge cooling). Note also because the oscillation ranges are 0.5 degrees, e_3 vs d parameters do not compare directly to Table 2 or Fig. 3, which are based on 0.2 degree oscillations.

Cryoprotectant		*a* (Å)	*c* (Å)	Volume (Å^3)	e_3 (°)	Slope (°/Å)	Intercept (°)
50% Xylose	Gas	96.53	106.45	991905	0.61	-0.005	0.63
		96.69	106.79	998375	0.59	-0.003	0.6
		96.67	106.82	998242	0.62	-0.006	0.64
		96.64	106.76	997063	0.62	-0.007	0.64
	Plunge	96.74	106.79	999408	0.70	-0.013	0.75
		96.7	106.70	997740	0.68	-0.014	0.73
		96.72	106.85	999556	0.69	-0.009	0.72
50% MPD	Gas	96.63	105.52	985278	0.65	-0.008	0.69
		96.7	105.77	989044	0.68	-0.015	0.73
	Plunge	96.89	106.14	996408	0.95	0.001	0.94
		96.65	105.48	985312	0.98	0.003	0.94
		96.76	105.71	989710	0.97	0.01	0.93
		96.64	105.67	986883	1.04	0.034	0.92
	Anneal	96.85	105.86	992959	0.86	0.005	0.85
		96.59	105.35	982876	0.66	-0.015	0.71
50% DMF	Gas	96.08	104.91	968463	0.94	-0.048	1.11
		96.25	105.30	975506	0.84	-0.028	0.95
	Plunge	96.35	105.27	977255	1.54	0.039	1.40
		96.15	105.16	972186	1.75	0.058	1.49
		96.23	105.28	974915	1.50	0.059	1.29
		96.29	104.95	973072	1.73	0.025	1.64
	Anneal	96.32	105.33	977204	0.72	-0.020	0.79
		96.27	105.26	975541	0.77	-0.022	0.84
		96.24	105.19	974284	0.76	-0.032	0.88
		96.39	105.24	977788	0.71	-0.005	0.63

Figure S1 Metrics for SAD structure determination of tetragonal thermolysin with Phenix. $CC_{1/2\text{-anom}}$ is for the low resolution bin (~30-8Å) from Aimless (Karplus & Diederichs, 2015, Evans, 2006). FOM is the figure of merit from Phenix using Autosol (Read & McCoy, 2011, Adams *et al.*, 2010). Rfree is for the autobuilt model in Phenix without any user intervention. Metrics are plotted against the overall redundancy of the data set to 2.0 Å, which corresponds to roughly 2x the anomalous redundancy. Blue dots = plunge cooled (protocol 3); solid orange = gas stream cooled (protocol 1); green dash = plunge cooled followed by rewarming and recooling in the gas stream (protocol 5).

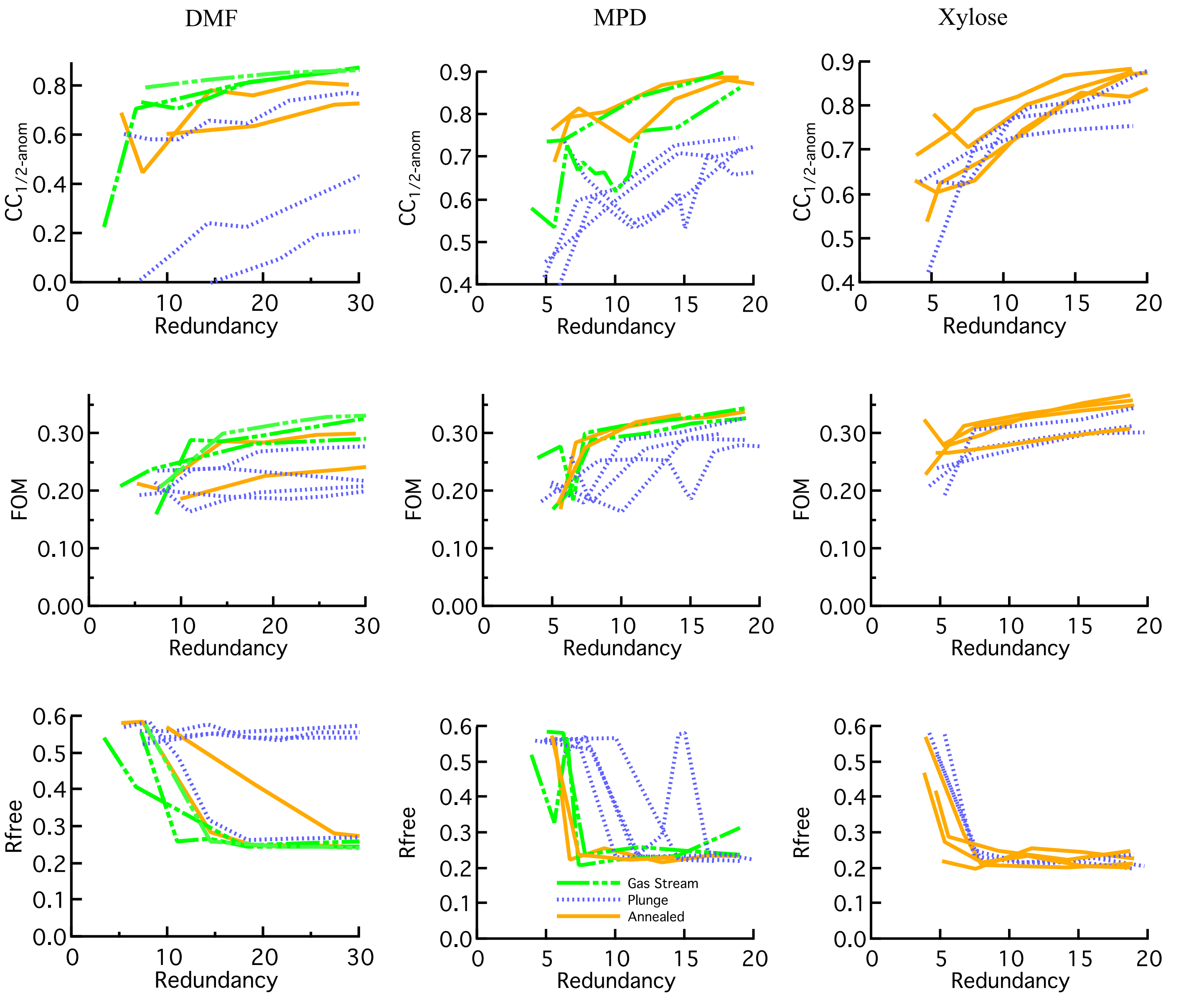

Figure S2 Number of tries for SAD substructure solution in ShelxD. For each run, 1000 or 100 tries were attempted. In cases where no solution was found, the trial number yielding the greatest value of CCall for 1000 tries is plotted as a solid circle. In cases where a solution was found, the trial number of the first solution is plotted. In general, fewer trials are required for gas stream cooling, for greater redundancy, and for xylose as cryoprotective agent.

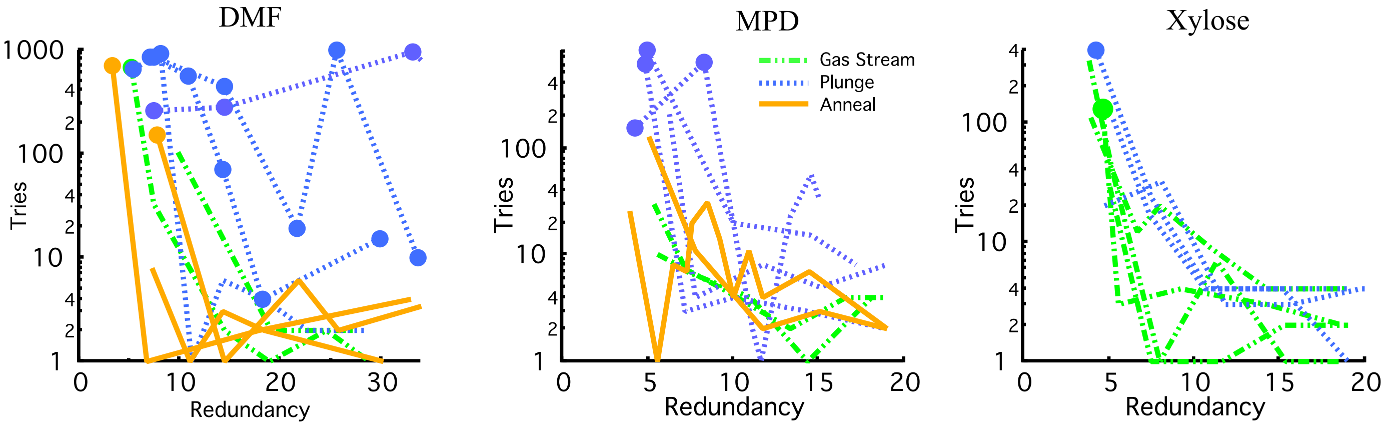

Figure S3 Plots of CCall (vertical axis) vs CCweak (horizontal axis) produced by ShelxD for tetragonal thermolysin crystals. The numbers listed in each frame indicate the overall data redundancy to 2.0 Å, and the resolution limit of the anomalous signal (using $CC_{1/2\text{-anom}}$ > 0.25 from ShelxC as the criterion for measurable anomalous signal). For ShelxD, the resolution of the data used was the lower of 3.0 Å and the resolution limit of the anomalous signal. The DMF plunged (mounting protocol 3) and DMF annealed (protocol 5) plots are from the same crystal mounted on the goniometer in approximately the same orientation. *For the DMF plunge cooled crystal, there appeared to be no measurable anomalous signal as judged from ShelxC for the 7.4 redundancy level, and 5.7 Å was used as the resolution cutoff for ShelxD. For the higher redundancy levels, $CC_{1/2\text{-anom}}$ vs resolution showed a minimum at ~4 Å resolution. The resolution quoted is that at which the $CC_{1/2\text{-anom}}$ first drops below 0.25. Using instead the higher resolution at which $CC_{1/2\text{-anom}}$ drops below 0.25 (3.3-2.9 Å) yields similar plots, but with smaller values of CCall and CCweak.

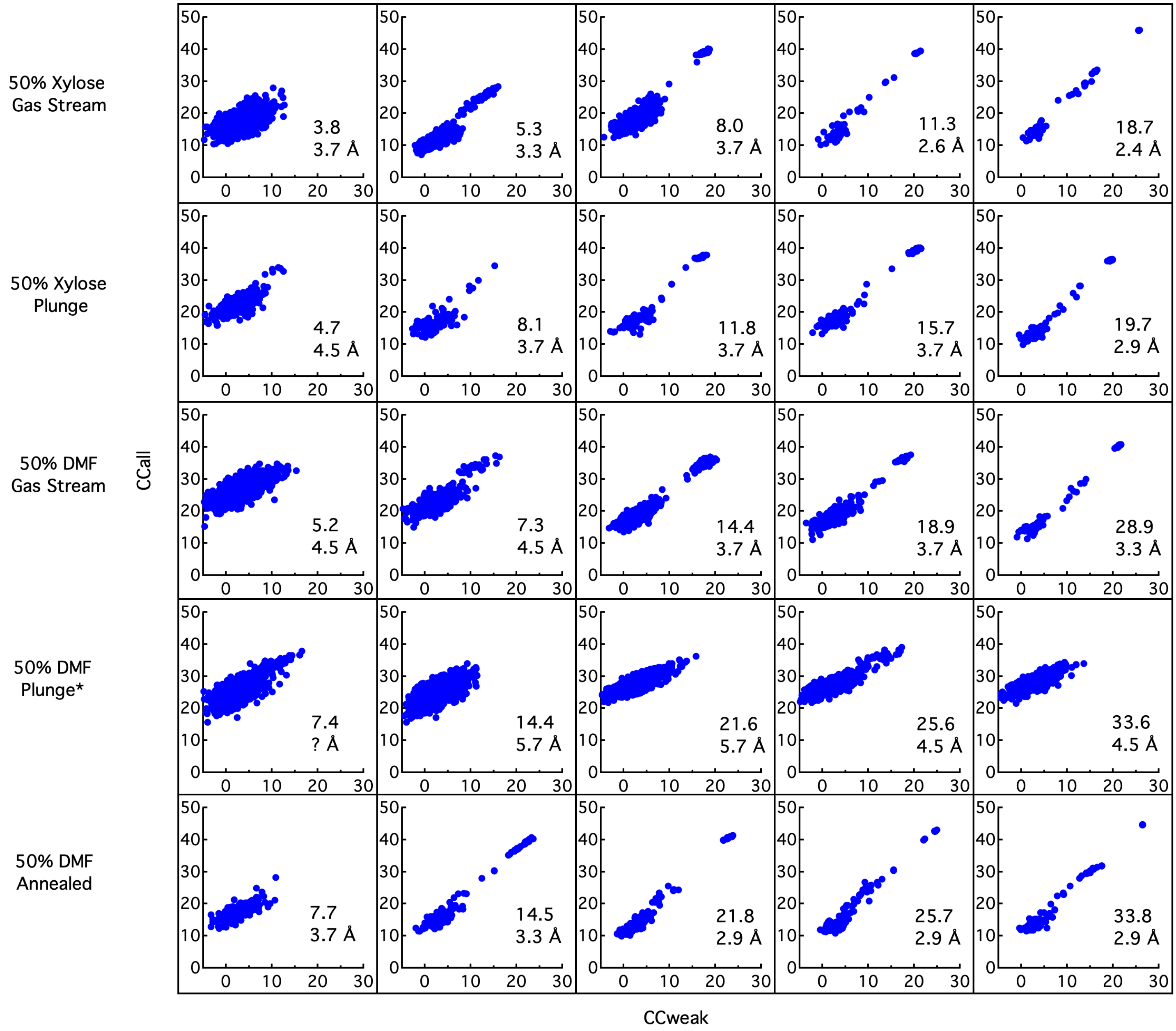

Figure S4 Plots of site occupancy in decreasing occupancy from ShelxD for the same crystals and data sets as Fig S3. B-factors are set to 20Å^2. The number shown in the graph is the overall redundancy to 2.0 Å resolution.

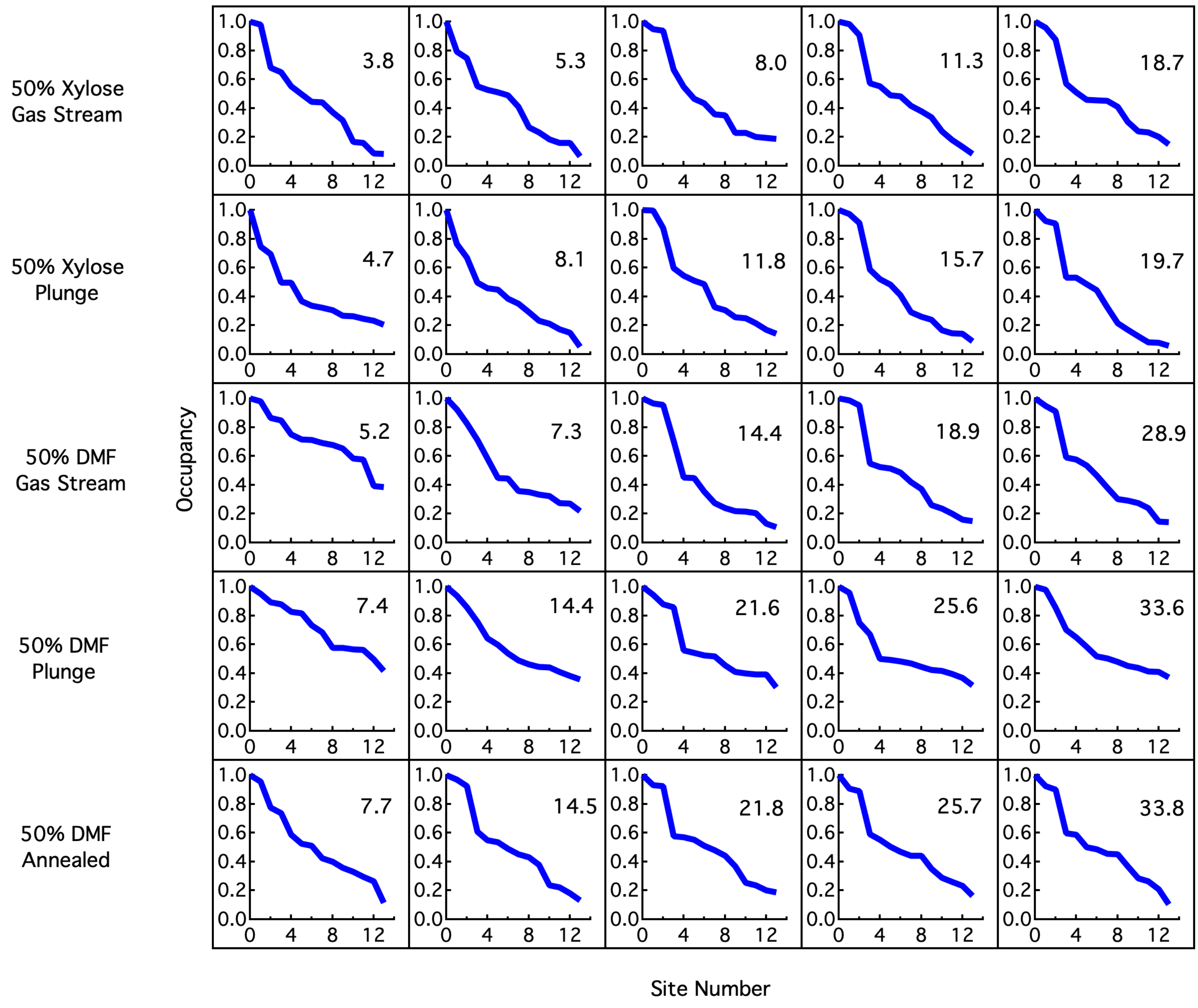

Figure S5 Plots of the reflecting range e_3 vs d for cryocooled tetragonal thermolysin crystals soaked in 50% DMF. Four different crystals were plunge cooled (protocol 3) indicated with blue dotted lines. Each plunge cooled crystal was then annealed (protocol 5), indicated with green dash-dot-dot lines. The thick dotted line corresponds to the one plunge cooled crystal whose structure could be determined via SAD. Two other crystals were cooled in the gas stream (solid orange lines – protocol 1). The positive slopes for the plunge cooled crystals indicate that the domain size was reduced with cooling. The negative slope of the annealed crystals indicates that the domain size then recovered with annealing. Similar improvement of the unit cell variation and/or domain misalignment is indicated by a reduction in the y-intercept of the plots with annealing. Values on the vertical axis are e_3 values averaged over the multiple runs used for the SAD data collection, and within the particular bin being plotted. Because the oscillation ranges were 0.5 degrees, these plots are not directly comparable to Table 2 or Fig. 3. See also Table S4.

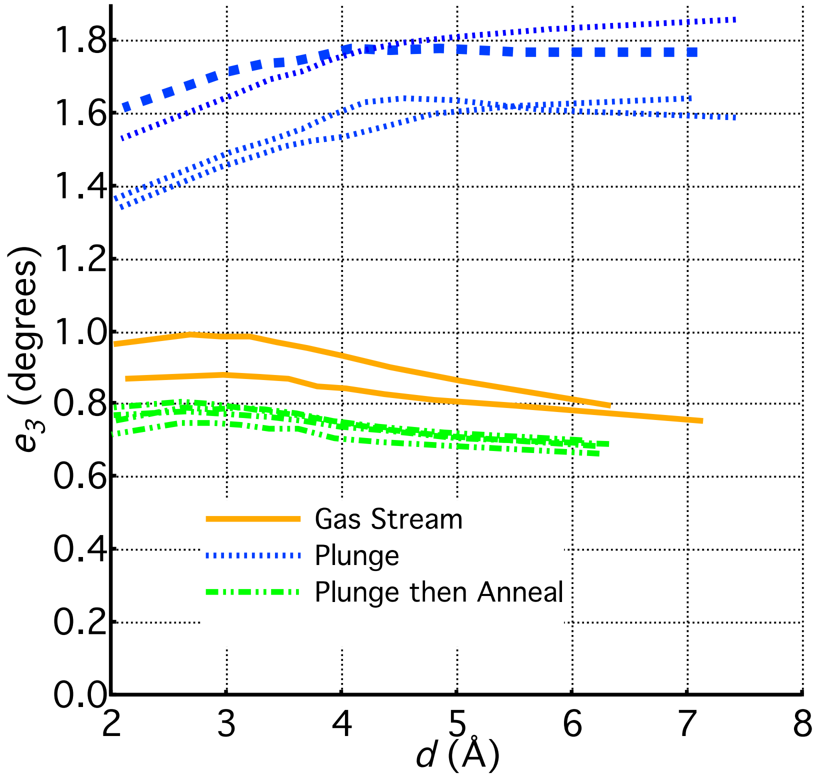

Figure S6 Correlation of side chain occupancies (left) and B-factors (right) between the plunge cooled and gas stream cooled structures of tetragonal thermolysin in the presence of 50% xylose. Data are shown for the 33 residues with alternative side chains. Linear fits have R-squared values of 0.73 and 0.95 for the Occupancy and B-factor data, respectively.

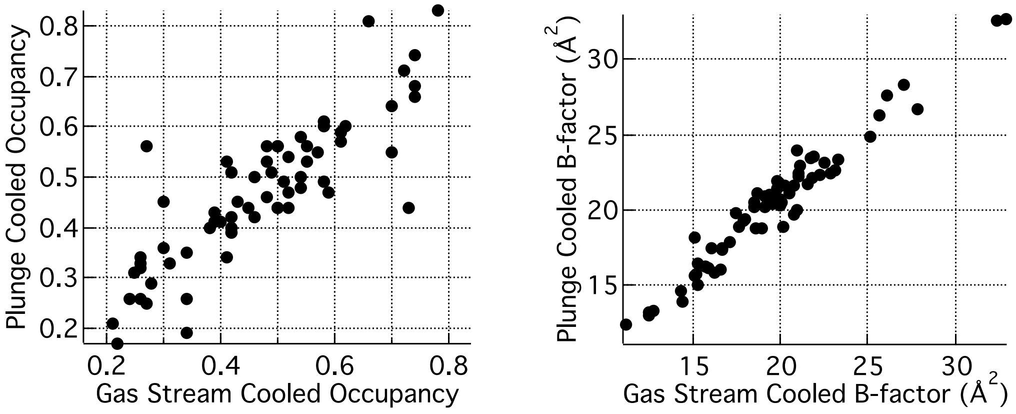

Figure S7

Side chain electron density similarity for tetragonal thermolysin cooled with different methods in the presence of 50% xylose. The plots show distributions of correlations between side chain electron density sampled about Chi-1 with Ringer (Lang *et al.*, 2010) for 252 residues (all non-alanine/glycine in 316 total residues in the structure). The plunge cooled and gas stream cooled structures show similar side chain electron density, with about the same level of departure from the RT structure. This suggests that cooling faster via plunging doesn't appreciably quench side chain conformations closer to the RT state than gas stream cooling. The RT structure was Protein Data Bank id 5UN3 (Juers *et al.*, 2018), for which X-ray data were collected on a crystal soaked in 50% xylose. The LT structures used in the plots are reported here.

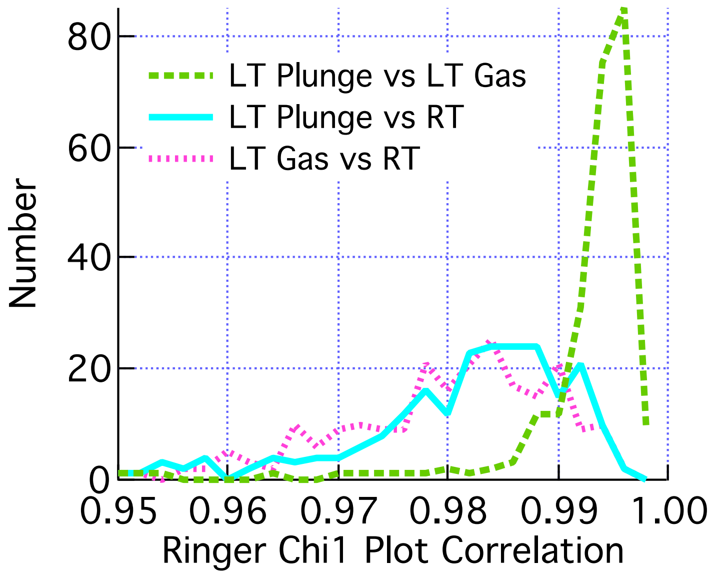

Supplement: Supplementary file 1 [file j-52-01222-sup1.pdf]
